# Inflammatory Bowel Disease and Obstructive Pulmonary Disease: A Two-way Association?

**DOI:** 10.7759/cureus.6836

**Published:** 2020-01-31

**Authors:** Azka Shahid Zergham, Amanpreet Kaur Sekhon, Amal Mebasher, Gantuya Tserenpil, Bilal Haider Malik

**Affiliations:** 1 Internal Medicine, California Institute of Behavioral Neurosciences and Psychology, Fairfield, USA

**Keywords:** inflammatory bowel disease, obstructive pulmonary disease, asthma, copd, crohn's disease, ulcerative colitis

## Abstract

Inflammatory bowel diseases (IBD) is an umbrella term that covers both ulcerative colitis (UC) and Crohn's disease (CD), which are chronic inflammatory conditions of the gastrointestinal system. Airway diseases are one of the most commonly studied manifestations of IBD. It is observed that populations with pre-existing obstructive pulmonary conditions are at higher risk of new-onset IBD. This newly documented evidence of increased incidence of IBD among patients with pulmonary diseases and the higher than the estimated prevalence of pulmonary diseases among IBD sufferers support the hypothesis of a two-way association. This review article focuses on summarizing the current knowledge and available evidence regarding the association between IBD and obstructive pulmonary diseases such as chronic obstructive pulmonary disease (COPD), emphysema, bronchiectasis, and asthma.

We utilized PubMed as the primary search source and database and included the free full-text articles available on it, published over the past five years. We reviewed literature from multiple regions of the world, such as the US, UK, China, and Canada and compiled this traditional review article utilizing the information collected from 4,966,459 patients. Specifications such as age and gender were not mentioned in all articles.

This review will serve to strengthen the existing research database concerning the relationship between IBD and obstructive pulmonary diseases. It will help to highlight the significance of the two-way association between IBD and obstructive pulmonary disease and the importance of treating these two conditions simultaneously. It will also raise awareness about the importance of timely detection of IBD and associated airway complications, leading to decreased disease burden and the treatment cost.

## Introduction and background

Inflammatory bowel disease (IBD) is a broad term that includes ulcerative colitis (UC) and Crohn's disease (CD), which are chronic inflammatory disorders of the digestive system [1]. The incidence and prevalence of IBD are steadily on the rise in Western as well as developing countries [2,3]. Patients with IBD are at an elevated risk of autoimmune and inflammatory conditions [4]. Airway diseases are one of the most commonly studied manifestations of IBD [5]. Concurrently, the risk of new-onset IBD is found to be higher in populations with obstructive pulmonary diseases such as obstructive pulmonary disease (COPD), asthma, and bronchiectasis compared to the general population without these conditions [6,7,8]. This newly documented evidence of IBD incidence among patients with pulmonary diseases and the higher than the estimated prevalence of pulmonary conditions among IBD patients support the hypothesis of a two-way association [5].

There are many factors in common between IBDs and obstructive pulmonary diseases, including the complex multifactorial basis, the chronic remitting-relapsing course of the disease, and the low-grade systemic inflammation [5]. Both obstructive pulmonary diseases (asthma, COPD, bronchiectasis) and IBDs arise through intricate interactions between genetic and environmental factors [6,7]. The similarities in the pathogenesis of these diseases, genetic susceptibility, and common environmental risk factors suggest that there is a mutual association between IBD and obstructive pulmonary diseases [7]. However, some literature also considers airway diseases merely as extraintestinal manifestations of IBD [5].

IBD and obstructive pulmonary diseases together account for substantial morbidity and a massive physical and economic burden on patients, families, and healthcare systems [6]. IBD and associated pulmonary comorbidities are clinically and scientifically crucial because much needs to be accomplished in the field to adequately address the physical, social, psychological, and economic challenges these conditions pose for the patient population. We wanted to explore the underlying factors leading to the relationship between these two conditions and also shed light on airway diseases as an extraintestinal manifestation of IBD [5]. We document the pathogenesis of these highly prevalent and multifaceted problems and also attempt to highlight the etiological basis of this association, the physical and psychosocial impact of the problem, and briefly discuss the pros and cons of conventional therapy versus alternative treatment [9]. We systematically review and summarize the latest database on PubMed concerning the co-occurrence of IBD and obstructive pulmonary diseases and the significant associated impact on public health and present this study to the scientific world [10].

We did not follow the Preferred Reporting Items for Systematic Reviews and Meta-analyses (Prisma) guidelines during the compilation of this traditional review article. PubMed was the main search source and database utilized. Regular keywords that yielded results on PubMed were "inflammatory bowel disease, obstructive pulmonary disease, asthma, COPD, Crohn's disease, and ulcerative colitis." Medical Subject Headings (MeSH) keywords used were "inflammatory bowel disease, asthma, and obstructive pulmonary disease."

We included the free full-text articles available on PubMed, which were published over the past five years. Older studies and databases other than PubMed were excluded. We tried our best to collect the data in an ethical and legal manner and to avoid plagiarism as much as humanly possible. We did not apply any quality assessment tools or undertake any statistical analysis while writing this traditional review article.

"Obstructive pulmonary disease, asthma, inflammatory bowel disease, COPD, Crohn's disease, and ulcerative colitis" were the regular keywords used, and they yielded 227,719, 184,269, 100,540, 84,028, 54,249, and 47,109 results, respectively, when searched on PubMed.

"Inflammatory bowel disease, asthma, and obstructive pulmonary disease" were the MeSH keywords used, and they yielded 30, 11, and 2 results, respectively, when searched on PubMed. Literature from multiple regions of the world, such as the US, UK, China, and Canada was reviewed. The compilation of this traditional review article utilized information collected from 4,966,459 patients. Specifications such as age and gender were not mentioned in all articles. No quality assessment tools were applied while writing this article.

## Review

A two-way association

The evidence of a genetic association between IBD and airway diseases and the complex interplay between these two diseases have been highlighted over the past years, but the idea of IBD development in patients with pre-existing pulmonary conditions has emerged quite recently [5]. This two-way hypothesis has been supported by various population-based studies that pinpoint the prevalence of IBD in patients with pulmonary diseases [5]. A similar population-based retrospective cohort study undertaken in Quebec, Canada assessed the incidences of UC and CD among patients suffering from obstructive pulmonary diseases [5]. This study indicates that women with asthma have a higher chance of developing CD as compared to men, and male COPD sufferers are more likely to present with UC than women [5]. Confirmation of these findings in broader studies and the emerging awareness of the association between airway diseases and IBD may play a significant role in the timely detection of IBD and the management of such patients. Future clinical research is needed to confirm this hypothesis of a two-way association between pulmonary disease and IBD [5].

Pathophysiology of the association

The loss of immune tolerance and subsequent immune disruption, which leads to increased responsiveness to environmental triggers, are the commonly described mechanisms in the case of both intestinal and airway diseases [11]. The intestine and airway are both derivatives of the same embryological structure and have similarities in their epithelial, glandular, and lymphoid tissues [12]. Patients with UC and CD usually show evidence of subclinical alveolitis on bronchoalveolar lavage and transbronchial sample biopsy [13,14]. In short, the common embryonic origin of the gut and respiratory epithelia and the suggested disturbance due to underlying immunological and environmental factors are considered to be the root causes of the association [11,12]. Studies suggest that a likely pathophysiological mechanism for the association between IBD and obstructive pulmonary disease is lung-gut cross-talk [15]. Respiratory associations of IBD were infrequently found in the discussed literature, and most of them lack a valid biological explanation [16]. However, a meta-analysis of several population-based studies showed that CD was associated with an increased risk of death by COPD, which is one of the most prevalent obstructive pulmonary diseases [17]. The quality of evidence regarding this association is moderate, and additional studies should be performed to determine a connection between these conditions and to explore the potential confounding impact of various environmental factors further [8].

Biochemistry of the association

A biochemical association between IBD and obstructive pulmonary disease has also come to light. It is postulated that an immunologic disruption between pro-inflammatory and anti-inflammatory cytokines can lead to a pro-inflammatory state [18,19]. Several studies in recent years have suggested a possible involvement of the gut flora and their role in the immune response [20,21]. Furthermore, autoantibodies produced against elastin or in response to certain antigens produced as a result of oxidative stress may play a role [20]. Interleukin-6 (IL-6) and transforming growth factor-beta (TGF-β) may collectively lead to T helper-17 (Th-17)-induced inflammation in multiple organs [21]. IL-13 is thought to direct abnormal natural killer cells and macrophage responses in both the intestine and the airway [22]. This aggregated data from multiple research articles support the viewpoint that the co-occurrence of IBD and pulmonary diseases have a shared multifaceted and complex biochemical and pathological base that encompasses oxidative stress and a hyperactive immune response [20,21,22]. South Korea has one of the highest incidence rates of IBD among all Asian countries [23,24,25,26]. Recent large-scale and scientifically robust studies regarding the aforementioned association, conducted in Asia, report findings comparable to regions with a high incidence of IBD such as Europe and Canada [7,16,27]. It is imperative to be up to date about the gastrointestinal symptoms suggestive of IBD in COPD patients. Thus, a well-designed observational cohort study comprising clinical data and other clinical variables is required to explore the biochemical association between COPD and IBD further [28].

Genetic basis of the association

The co-occurrence of IBD and airway diseases suggests that there might be a genetic basis to the association. Genome studies have revealed a convergence of areas of gene linkage for IBD and asthma [29]. Distinct genetic loci, including mothers against decapentaplegic homolog 3 (SMAD3), DENN domain containing 1B (DENND1B), and Solute carrier family 22, member 4/5 [SLC22A4/5 (5q31/IBD5)], are known to be associated with both CD and asthma [30]. On the other hand, the ORMDL sphingolipid biosynthesis regulator 3 (ORMDL3) gene, which is present in both CD and UC, is also seen in childhood-onset asthma. The relationship between nucleotide-binding oligomerization domain-containing protein 2 (NOD2) gene polymorphism leads to the advancement of both CD and COPD [31,32]. This favors the hypothesis that there is a shared genetic susceptibility underlying the association between IBD and airway diseases. NOD2 proteins bring about the recognition of peptidoglycan bacterial wall, leading to bacterial antigen recognition, which serves as a precursor of immune defense activation [32]. Some studies in the pediatric population also postulate that genes such as zona pellucida binding protein 2 (ZPBP2) and pyrin and HIN domain-containing protein 1 (PYHIN1) are responsible for the relationship between pediatric IBD and autoimmune conditions, including pulmonary diseases [33]. In short, this recent data support the idea that there is a common genetic basis and overlap between regions coding for IBD and obstructive pulmonary diseases [29,33].

Pulmonic manifestations of IBD therapy

It is very challenging to differentiate between the pulmonary manifestations of IBD and drug-related lung disease [34]. Sulfasalazine has been employed as a mainstay therapy for IBD for the past 60 years. Sulfasalazine-related pulmonary side effects are infrequent, and the literature review provided just 50 cases of pulmonary toxicity induced by sulfasalazine [35]. Only two isolated cases of interstitial fibrosis have come to light since 1972 [35,36]. Pulmonary symptoms in these patients resolved after the discontinuation of the drug. Similarly, mesalazine, which is a 5-aminosalicylic acid derivative, is also commonly employed to treat IBD. A few cases of organizing pneumonia (OP) related to mesalazine have been reported so far, and mesalazine-related lung diseases include OP, eosinophilic pneumonia, and interstitial pneumonia. These case reports serve to remind the clinicians to be mindful of possible side effects of IBD therapy and extraintestinal manifestations of the disease [34]. Moreover, the use of anti-tumor necrosis factor-α (anti-TNF-α) agents for the treatment of IBD is also increasing. A study undertaken in India re-confirms that there is a high chance of reactivation of pulmonary TB with the use of infliximab for the treatment of IBD in TB-endemic areas, including in India. This study is, however, limited by a small sample size, but even this small sample size has yielded clinically significant results [37]. To sum up, it is imperative for the physicians to distinguish between the pulmonic effects of IBD and lung conditions arising secondary to the pharmacological management of IBD [34].

Effect of cigarette smoke on the association

Cigarette smoke is considered a significant risk factor for both COPD and CD [16]. The subsequent development of COPD in smokers further increases the risk of CD. COPD patients and cigarette smokers have 2.72 and 2 times higher risk for CD, respectively [16,38]. Smoking is associated with increased disease severity and relapse in CD patients [15]. Recent studies postulate that COPD patients have higher permeability of the intestinal barrier, which is associated with decreased lung function, both at rest and during exercise. However, minimal experimental data can be found to support these hypotheses [38-42]. The literature review regarding the association between IBD and obstructive pulmonary disease does suggest that chronic inhalation of cigarette smoke and subsequent development of COPD brings about pathophysiologically relevant changes in the colon and ileum, which increase chances of IBD occurrence. Most of the current studies with scientifically relevant results have been performed on mouse models. Broader spectrum clinical trials regarding the effect of cigarette smoke and environmental factors are required [16].

Recent developments

A review of the literature reveals that there is an intricate association between IBD and airway diseases. Newer evidence suggests that not only can airway diseases develop as an extraintestinal manifestation of IBD, but also IBD can have a higher rate of occurrence in airway disease patients [5]. However, this pulmonary dysfunction is not directly related to the severity of IBD. Sometimes, airway changes are subtle. Physicians should consider pulmonary function testing for all IBD patients, specifically the ones having increased erythrocyte sedimentation rate (ESR) levels or any respiratory symptoms to prevent further pulmonary damage. Other noninvasive methods to gauge pulmonary function are under consideration. These include induced sputum (cytological analysis) and detection of exhaled air (nitric oxide, carbon monoxide, and markers of oxidative stress). This will encourage broader studies with a large sample size on IBD patients and may facilitate a better understanding of the pathogenesis of the IBD-related pulmonary diseases and the association between IBD and obstructive pulmonary conditions [43].

A recent significant population-based study highlights that new-onset IBD increases the risk of mortality in patients suffering from COPD and asthma-associated COPD. This study also substantiates the previously mentioned association between IBD and COPD. It also indicates that IBD may be one of the comorbidities contributing to the increased mortality rate in COPD patients. Hence, it is also imperative to perform IBD assessment in COPD patients, and failure to do so could lead to delayed diagnosis and treatment [44].

## Conclusions

This review article aimed to explore the current knowledge about the suggested two-way association between IBD and obstructive pulmonary disease. We have come across many articles supporting the pathophysiological, genetic, biochemical, pharmaceutical, and environmental basis of this association. Pulmonary manifestations of IBD have been under consideration for a long time. Mainstream IBD therapy has also been found to have adverse consequences on the pulmonary system. Our paper will serve to increase awareness about the association between IBD and obstructive pulmonary disease and the importance of tackling these two conditions simultaneously. Timely detection of IBD and associated airway complications will play a significant role in decreasing the disease burden and the treatment cost. After careful review and analysis of recent data, we can confidently conclude that a strong association exists between IBD and obstructive airway diseases. However, broader studies with large sample sizes are required to explore this association further. A better understanding of the underlying pathogenesis and relevant environmental factors is needed to introduce treatment and medication options that are sustainable and cost-effective with low side-effect profiles.
